# Oscillatory Activities in Neurological Disorders of Elderly: Biomarkers to Target for Neuromodulation

**DOI:** 10.3389/fnagi.2017.00189

**Published:** 2017-06-13

**Authors:** Giovanni Assenza, Fioravante Capone, Lazzaro di Biase, Florinda Ferreri, Lucia Florio, Andrea Guerra, Massimo Marano, Matteo Paolucci, Federico Ranieri, Gaetano Salomone, Mario Tombini, Gregor Thut, Vincenzo Di Lazzaro

**Affiliations:** ^1^Clinical Neurology, Campus Biomedico University of Rome Rome, Italy; ^2^Nuffield Department of Clinical Neurosciences, University of Oxford Oxford, United Kingdom; ^3^Department of Clinical Neurophysiology, Kuopio University Hospital, University of Eastern Finland Kuopio, Finland; ^4^Centre for Cognitive Neuroimaging (CCNi), Institute of Neuroscience and Psychology, University of Glasgow Glasgow, United Kingdom

**Keywords:** neuromodulation, non-invasive brain stimulation, oscillations, TMS/tDCS, EEG

## Abstract

Non-invasive brain stimulation (NIBS) has been under investigation as adjunct treatment of various neurological disorders with variable success. One challenge is the limited knowledge on what would be effective neuronal targets for an intervention, combined with limited knowledge on the neuronal mechanisms of NIBS. Motivated on the one hand by recent evidence that oscillatory activities in neural systems play a role in orchestrating brain functions and dysfunctions, in particular those of neurological disorders specific of elderly patients, and on the other hand that NIBS techniques may be used to interact with these brain oscillations in a controlled way, we here explore the potential of modulating brain oscillations as an effective strategy for clinical NIBS interventions. We first review the evidence for abnormal oscillatory profiles to be associated with a range of neurological disorders of elderly (e.g., Parkinson’s disease (PD), Alzheimer’s disease (AD), stroke, epilepsy), and for these signals of abnormal network activity to normalize with treatment, and/or to be predictive of disease progression or recovery. We then ask the question to what extent existing NIBS protocols have been tailored to interact with these oscillations and possibly associated dysfunctions. Our review shows that, despite evidence for both reliable neurophysiological markers of specific oscillatory dis-functionalities in neurological disorders and NIBS protocols potentially able to interact with them, there are few applications of NIBS aiming to explore clinical outcomes of this interaction. Our review article aims to point out oscillatory markers of neurological, which are also suitable targets for modification by NIBS, in order to facilitate in future studies the matching of technical application to clinical targets.

## Introduction

Oscillatory activities in neural systems may play a functional role (Gray, 1994) and abnormalities in neural synchronization mechanisms might be involved in the pathophysiology of several neuropsychiatric disorders (Uhlhaas and Singer, 2006). Thousands of neurons synchronize their activity to generate a typical oscillatory pattern that can be measured either through an electroencephalogram (EEG) from scalp electrodes or through local field potentials (LFPs) or intracranial EEG recordings from small-sized, implanted electrodes in the brain. The recording of the magnetic field induced by the same activity is referred to as magnetoencephalography (MEG). A common obstacle in interpreting these signals arises because a given macroscopic extracellular signal can be generated by diverse cellular events. Indeed, deriving macroscopic variables from their elementary causal constituents requires to solve the inverse problem (Nunez and Srinivasan, 2006). As a consequence, explaining the physiology of neural oscillations at the macroscale is complex because each rhythm is the resultant of the physiology of specific neural assemblies, in particular a mix of their spontaneous and evoked activity (Buzsáki et al., 2012). Many non-invasive and invasive studies with a clinical focus described abnormal oscillatory activities in different neurological disorders typical of elderly patients, such as Parkinson’s disease (PD), stroke, dementia and epilepsy. However, it is still unclear whether these abnormalities have a pathophysiological role, and by extension, whether these disorders can be considered “oscillopathies”, or whether they represent merely epiphenomena of the neural changes causally underlying the symptoms.

Non-invasive brain stimulation (NIBS) techniques are able to induce functional changes in the brain, by inducing and modulating ongoing oscillatory activity (Krawinkel et al., 2015). Thus, these techniques might have a potential role in both the diagnosis and treatment of neurological disorders, revealing an abnormal oscillatory response, and/or be of use in the attempt to rebalance the activity in abnormally functioning neural circuits.

The aim of this review article is two-fold: to (1) critically analyze spontaneous and evoked neural oscillations in neurological disorders of elderly in terms of their possible pathophysiological role; and (2) evaluate the effects of NIBS on these neuronal oscillations and their possible therapeutic promise.

## Spontaneous and Task-Related Oscillatory Activity

Brain connectivity is a dynamic process, which changes with aging, and can be modulated by cognitive and physical training (Bamidis et al., 2014). Different groups of neurons tend to synchronize their activity at specific frequencies, defined as rhythms, which have been categorized in five canonical frequency bands: delta (<4 Hz), theta (4–8 Hz), alpha (8–12 Hz), beta (12–30 Hz) and gamma (30–90 Hz). Higher frequencies (>90 Hz) have been subsumed as high-frequency oscillatory (HFO) activity (Schomer and Lopes da Silva, 2012). EEG can easily detect oscillations from the delta to the beta band, while it is less suited for recordings of gamma and HFO activity, since the signal intensity of these activities in EEG does not emerge from the abundant artifactual activity (mainly muscular and surrounding direct current) of the scalp. The limited influence of muscular activity on MEG and intracranial recordings render these two techniques suitable to analyze also very high frequency oscillations (HFO). In the following sections, we will survey the existing literature as to evidence for a physiological and pathophysiological role of these different rhythms.

### Delta

**Figure 1:**
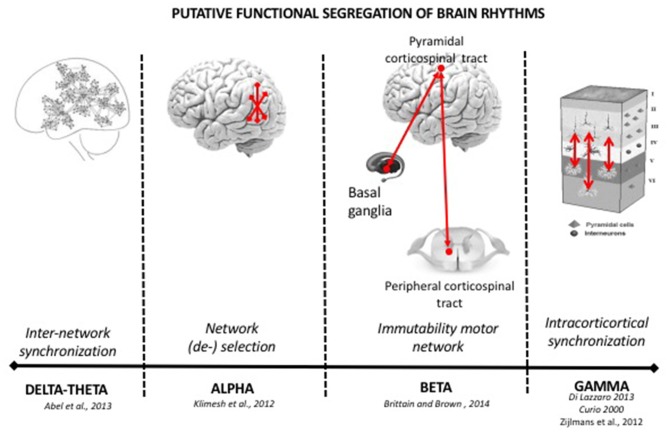
Functional role of brain rhythms in healthy subjects. In healthy participants, brain oscillations are subsumed in specific canonical frequency band, with presumably different functions. Slower rhythms (delta-theta) are associated with higher power spectra generated by the synchronization of a big number of neurons or of networks of neurons. They prevail during sleep, when memory consolidation phenomena occur. The alpha rhythm is the dominant oscillation in the awake state has been associated with inhibitory functions to gate information flow. Beta activity is a rhythm of the motor system (pyramidal and extra-pyramidal) and inhibits the changing of motor activity. Gamma and higher oscillations are resident in intracortical activity synchronizing small group of neurons. See the text of manuscript for more details.

In physiological conditions, delta activity is the most prominent EEG feature of human non-rapid eyes movement (NREM) sleep. It originates in cortical neurons and has been proposed as possible mediator of sleep-dependent synaptic plasticity (for a review, see Tononi and Cirelli, 2012), synchronizing the excitability state of huge groups of cortical neurons to facilitate cortico-hippocampal memory processes (Abel et al., 2013; Figure 1). During wakefulness, delta activity is almost absent in physiological conditions, but it appears both after subcortical brain lesion sparing cerebral cortex (Gloor et al., 1977; Steriade et al., 1993, 2001) and after the induction of cortical plasticity (Assenza et al., 2013a). Clinical studies in acute stroke patients suggest that delta activity of the affected hemisphere (AH) is related to both the lesion volume and the acute neurological deficit (Assenza et al., 2009), and that its spreading from the AH to the unaffected hemisphere (UH) is associated with poor prognosis (Finnigan et al., 2004). Advanced EEG analyses demonstrate that delta activity of the UH results from an interhemispheric communication breakdown of electrical signals between the two hemispheres and that this might, in turn, interfere with the UH contribution to recovery, i.e., plasticity processes (Graziadio et al., 2012; Assenza et al., 2013b). Patients with focal epilepsy show an increase in delta activity during daytime and sleepiness, but its biological meaning is uncertain (Pellegrino et al., 2017).

### Theta

Human EEG experiments reliably demonstrated the relevance of frontal (Klimesch, 1999) and hippocampal (Colgin, 2016) theta power in memory tasks. One theory posits that items presented according to a theta rhythm can induce Hebbian plasticity and thus long-term potentiation (LTP) or depression favoring memory retention (Jensen and Lisman, 1996). More specifically, LTP is generated by the activation of slow NMDA channels, which own a 150 ms time constant. Therefore, repetitive activity of these channels can produce theta oscillations supporting episodic memory (Jensen and Lisman, 1996). Others have suggested that cortical and hippocampal theta activity guides cortico-hippocampal synchronization to realize consolidation processes in NREM and REM sleep phases (Abel et al., 2013; Figure 1). Clinical studies show that theta band power increases typically after acute brain lesions. Activity in this frequency band is very sensitive to acute neural damage induced by perfusion reduction (Astrup et al., 1979). However, its role in neural disorders is controversial, as in sub-acute and chronic stages after stroke, the persistence of theta oscillations can be a sign of a damaged network or alternatively of its attempt to reorganize itself, in analogy to delta activity (Assenza et al., 2013b). Accordingly, the enhanced presence of this slow oscillation may suggest a network disassembly, but also signal a role in promoting plasticity in the context of neural network reorganization.

### Alpha

Alpha-band activity is the dominant oscillation in the awake human brain. It is prominently observed over areas of the visual and attention network, where it is negatively related to visual perception (Hanslmayr et al., 2005; Thut et al., 2006), which is in line with the hypothesis that this frequency band plays an inhibitory role (Klimesch et al., 2007). Conversely, posterior alpha-band activity over visual/attention areas is positively related to non-perceptual functions such as memory (Hanslmayr et al., 2005; see also Jokisch and Jensen, 2007). This suggests that the inhibition of external (visual) input is helping performance of internal (memory) tasks, and has led to the hypothesis that alpha activity may shape functional network architecture through inhibiting task-irrelevant areas. A further relevant feature of alpha-band activity is that it is the only rhythm (with the exception of slow beta activity) that can respond to a stimulus and/or task demand by either decreasing or increasing its amplitude/power, namely showing event-related desynchronization (ERD) and event-related synchronization (ERS; Klimesch, 2012). More specifically, brain regions that are activated during a task exhibit ERD, whereas regions associated with processing of irrelevant and/or potentially interfering tasks exhibit ERS (Pfurtscheller and Lopes da Silva, 2004). Such opposite changes in posterior alpha-power (i.e., ERD vs. ERS) have been observed with tasks varying in stimulus modality (e.g., visual vs. sounds), stimulus processing domain (color vs. motion) or stimulus side (left vs. right), depending on which sensory feature needs to be processed or suppressed, and on the areas engaged in the processing of the relevant or irrelevant feature (Foxe and Snyder, 2011). This supports the theory that alpha ERS is the EEG correlate of cortical inhibition, while alpha ERD is a reduction of this inhibition. In brief, alpha activity may promote selection of cortical networks by inhibiting task-irrelevant areas (Pfurtscheller and Lopes da Silva, 2004; Klimesch, 2012; Figure 1).

In cognitive disorders, the corruption of the alpha band activity is a prominent finding. In Alzheimer’s disease (AD), there is strong evidence in favor of impaired resting state cortical alpha activity. In comparison to healthy age-matched controls, AD patients show a decrease of posterior alpha power along with a significant anterior shifting of the maximum alpha peak, mainly during oscillatory activity at rest (Huang et al., 2000; Babiloni et al., 2004, 2013a; Jeong, 2004). Notably, this decrease in alpha band activity directly correlates with the cognitive deficits and the severity of the disease (Jeong, 2004). Moreover, longitudinal follow-up of those patients reveals that the aforementioned changes are short-term predictors of progression from Mild Cognitive Impairment to dementia (Jelic et al., 1996, 2000; Huang et al., 2000; Rossini et al., 2006). In contrast, the reduction of parietal-occipital alpha-beta power is a marker of dementia progression (Coben et al., 1985; Soininen et al., 1989, 1991). Furthermore, a constant and reliable hallmark of AD across EEG and MEG studies is the significant decrease of coherence at alpha frequency in temporo-parietal areas (Leuchter et al., 1992; Locatelli et al., 1998; Wada et al., 1998; Jelic et al., 2000; Adler et al., 2003; Montez et al., 2009). Similarly, in stroke patients, a prominent ipsilesional alpha-decrease is a predictor of poor outcome (de Vos et al., 2008) and conversely its preservation is a marker of good prognosis. In unilateral middle/anterior cerebral artery ischemic stroke, the alpha band decrease is more prominent in the brain regions responsible for those behavioral deficits that will still be present 3 months after stroke (van Putten and Tavy, 2004). Moreover, a high ratio of delta/alpha power during subacute stroke is associated with higher scores of NIHSS at 30-days post-stroke. The relevance of alpha rhythm in clinical symptoms has also been highlighted in extrapyramidal disorders. After levodopa administration in PD patients, alpha-activity is increased in the pedunculopontine nucleus (PPN), bidirectionally-coupled with similar changes in cortical EEG, when participants perform self-paced movements (Androulidakis et al., 2008). These findings suggest a possible physiological role of these oscillations in the PPN area, such as promoting motor related attentional processes, which can be affected in non-treated PD.

### Beta

#### Beta Activity in the Cortico-Spinal System

Voluntary movements, or even cues that predict the need for a voluntary movement, are preceded and accompanied by suppression of the beta rhythm (Pfurtscheller and Lopes da Silva, 2004). However, the motor relevance of beta-activity is not confined to movement coding, but becomes also evident when a voluntary isometric contraction is sustained (Brown and Marsden, 1998). In this task, cortical beta activity synchronizes with electromyographic oscillations, as evidenced in the so-called cortico-muscular coherence. During a sensorimotor task, as in isometric muscle contraction, cortico-muscular coherence can also recruit primary sensory areas, for which synchronized oscillatory activity in the beta band correlates with performance (Tecchio et al., 2008; Chakarov et al., 2009). Thus, beta band is a prominent rhythm of the corticospinal system, tracking the efficient flow of motor information between the cortex and the periphery.

Clinical modulation of beta-activity occurs in acute and chronic, vascular and degenerative pyramidal system lesions. Compared to healthy controls, acute and chronic ischemic stroke patients with motor deficits have lower bi-hemispheric beta activity (EEG and MEG studies; Tecchio et al., 2005; Dubovik et al., 2012; Graziadio et al., 2012) and reduced beta ERS after a somatosensory input (Rossiter et al., 2014). While a more prominent ERS reduction in the AH is associated with bigger lesions (Laaksonen et al., 2012), the preservation of beta activity in both hemispheres in the acute phase correlates with a better motor outcome (Tecchio et al., 2005, 2007). In addition, beta ERS after a tactile stimulation is typical of patients with greater motor dexterity (Gerloff et al., 2006) and the increase of beta ERS in the AH from acute phase to chronic phase predicts hand dexterity recovery (Laaksonen et al., 2012). Patients affected by amyotrophic lateral sclerosis (ALS) show a significantly smaller beta ERD compared to controls during motor imagery as revealed by EEG studies in ALS patients with an emphasis on brain computer interface technology (Kasahara et al., 2012). Furthermore, ALS patients exhibit reduced beta ERS after movements (Riva et al., 2012) which in addition is merely unilaterally localized (beta rebound), as compared to a bilateral phenomenon in controls (Bizovičar et al., 2014). These results are in line with alterations of the beta rhythm observed in stroke patients with motor deficits. Because observed in a pathophysiological setting purely affecting the pyramidal system, this confirms the relevance of oscillatory beta activity in pyramidal tract physiology and pathology.

#### Beta Activity in Cortico-Basal Ganglia Loops

There is wide agreement on the association of beta activity in cortico-basal ganglia loops (13–30 Hz) with static motor control, such as tonic or postural contraction (Jenkinson and Brown, 2011). In PD, in the absence of levodopa therapy, the cortical-subcortical motor loops tend to synchronize within the beta band. After levodopa treatment however, they tend to synchronize within higher frequencies (>70 Hz; Brown et al., 2001; Williams et al., 2002; Foffani et al., 2003). Deep brain stimulation (DBS) at 20 Hz (beta band) of the subthalamic nucleus (STN) synchronizes GP internus (GPi) at the same frequency, whereas high frequency (>70 Hz) STN DBS suppresses beta band GPi oscillations (Brown et al., 2004). In line with (in)direct modulation of these oscillations having a clinical effect, STN or Gpi high frequencies stimulation improves PD motor symptoms (Brown et al., 2004), while beta frequency stimulation of STN has an antikinetic effect in PD patients (Timmermann et al., 2004; Fogelson et al., 2005; Chen et al., 2011). Levodopa administration, which is effective in treating bradykinesia, decreases basal ganglia beta oscillation (Kühn et al., 2005, 2009; Weinberger et al., 2006; Ray et al., 2008; Zaidel et al., 2010). Conversely, anticholinergic drugs that are more effective in treating tremor do not affect basal ganglia beta band activity (Priori et al., 2004). Furthermore, cortical beta oscillations are inversely correlated with movement acceleration (Gilbertson et al., 2005). Thus, it can be argued that enhanced basal ganglia beta band activity may be an indirect marker of bradykinesia, adjustable by levodopa therapy (Brown et al., 2001; Levy et al., 2002; Priori et al., 2004; Brown and Williams, 2005). The relevance of beta band in basal ganglia motor control is corroborated by data on dystonia, where the GPi LFP presents a lower beta band power than in PD (Silberstein et al., 2003). Therefore, excessive beta synchronization in the basal ganglia circuit has an antikinetic effect, as occurs in untreated PD patients, while its reduction with treatment leads to bradykinesia improvement, but excessive reduction can lead to hyperkinetic disorders, such as dystonia.

Besides mere motor control, a modulation of beta-band (specifically, an increase in functional connectivity between bilateral occipital, parietal, temporal and prefrontal regions) was also observed after a mixed physical-cognitive training in elderly patients with mild cognitive impairment (Klados et al., 2016).

In conclusion, in the pyramidal and extra-pyramidal motor network, brain oscillations play a key role in motor control, with beta activity reflecting a residential rhythm of the motor system, necessary for its proper functioning. Beside its antikinetic role, beta activity should be considered in a wider context of information gating favoring the maintenance of the status quo of the selected neuronal system (pyramidal and extra-pyramidal; Engel and Fries, 2010; Brittain and Brown, 2014; Figure 1).

### Gamma and High Frequency Oscillations

Because of the broad frequency spectrum covered by Gamma activity and HFOs, there are many types of oscillations in these higher frequency bands (>30 Hz). Those in the normal brain appear to facilitate synchronization and information transfer necessary for cognitive processes, memory and sensory-motor integration (Tecchio et al., 2008; Vinck et al., 2013), while other classes of HFOs reflect fundamental mechanisms of epileptic phenomena and of basal ganglia movement disorders in patients. We will focus on the latter as the aim of the present article is to provide basic neurophysiological background in a clinical perspective.

As discussed above, in PD patients on levodopa therapy, the cortico-subcortical motor network tends to synchronize into gamma and higher frequencies (Brown et al., 2001, 2004; Williams et al., 2002; Foffani et al., 2003). Moreover, while LFPs recordings from the STN of dopamine treated PD patients show a dopamine- and movement-dependent 300-Hz rhythm (Foffani et al., 2003), no consistent rhythm is found in the absence of dopaminergic medication at rest in the 100–1000 Hz frequency band in most cases. More specifically, levodopa or apomorphine administrations elicits a 300-Hz rhythm, which is modulated by voluntary movements. The dopamine-dependent 300-Hz rhythm therefore probably reflects a bistable compound of STN activity supporting high-resolution information processing in the basal ganglia circuit. The switching from low to high frequencies oscillations (>300 Hz), has a neurophysiological prokinetic role, related to the motor improvement that follow levodopa treatment in PD. Furthermore, the absence of subthalamic 300-Hz may represent a pathophysiological clue in PD and thus also provide the rationale for an excitatory and not only inhibitory use of DBS mechanisms of action in patients (Foffani et al., 2003; Özkurt et al., 2011).

In epilepsy, pathognomonic oscillatory activity can be subsumed in two highly specific patterns: the spike-wave complex (SWC), and HFO. The SWC is the EEG marker of the paroxysmal depolarization shift occurring in neuronal cells. It is maximally expressed in absence epilepsy, where 3 Hz SWCs are reverberating in the thalamo-corticothalamic loop and cause a breakdown of all higher cognitive functions (Niedermeyer and Lopes da Silva, 2005). HFOs on the other hand are generated locally (as mechanisms must be fast enough to synchronize activity within 2 ms to 5 ms) and are frequently observed in epileptic patients. Candidate mechanisms of HFOs are ephaptic interactions, electrotonic coupling via gap junctions, or fast synaptic transmission (Zijlmans et al., 2012). HFO activity has recently been proven to be an excellent biomarker for the epileptogenic zone (Zijlmans et al., 2012), and can be sub-classified in ripples (80–250 Hz) and fast ripples (250–600 Hz; Bragin et al., 1999; Jirsch et al., 2006). Interictal HFOs mostly occur during slow wave sleep. In epilepsy surgery, removal of tissue with HFOs seems to predict good surgical outcome, even better than removal of the ictal onset zone (Jacobs et al., 2010), indicating that HFOs may mark cortex that needs to be removed to achieve seizure control. In brief, both HFO and SWC are excellent markers of epileptic disease activity.

## Oscillatory Activities Evoked by Stimulation of The Pheripheral and Central Nervous System

In addition to examining intrinsic spontaneous and task-related brain oscillations across disorders, electrophysiology can be used to probe oscillatory brain responses and reverberations provoked by external stimuli such as tactile stimuli presented to the hand, the presentation of visual stimuli or transcranial magnetic stimulation (TMS) pulses applied directly to the cortex. This approach is routinely used for diagnostic purposes (see e.g., sensory or motor evoked potentials, i.e., SEPs or MEPs) in various peripheral and central nervous system diseases. More recently, the approach has been flagged to be of interest also for the characterization of network organization in the normal and dysfunctional brain, in particular when single pulse TMS is used over different cortical area in combination with multichannel EEG recordings (e.g., Rosanova et al., 2009; Bortoletto et al., 2015).

### Sensory-Motor Cortex Stimulation

To assess the integrity of the ascending and descending tracts and cortical projection zones, the sensorimotor cortex can be stimulated by either direct electrical or magnetic stimulation or by peripheral nerve stimulation, and the evoked activity can be recorded from the cerebral cortex or higher cervical segments using spinal electrodes located close to the axons of the corticospinal tract.

**Figure 2:**
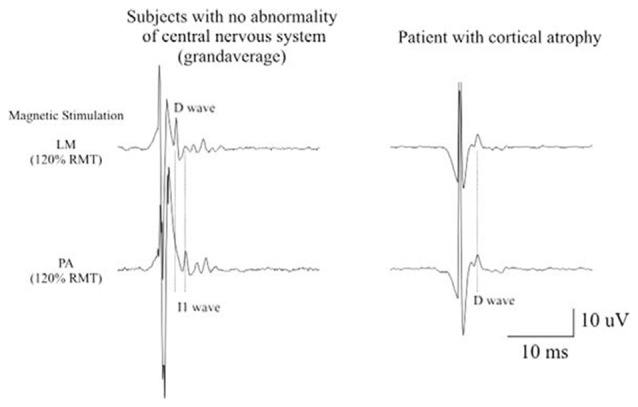
Epidural activity recorded in a patient with cerebral cortex atrophy. Descending volleys evoked by latoro-medial magnetic stimulation and posterior-anteriormagnetic stimulation at 120% resting motor threshold (RMT) in five patients with no abnormality of the central nervous system and at the maximum stimulator output in one chronic alcoholic patient. The grand averages of epidural volleys recorded in patients with no abnormality of central nervous system are shown on the left and the averages of epidural volleys (of 10 sweeps) recorded in the patient with cerebral cortex atrophy are shown on the right. The latencies of the D and I1 waves evoked by LM and PA magnetic stimulation are indicated by vertical dotted lines. In control subjects, LM stimulation evokes a large D wave followed by 5 I waves; PA stimulation evokes only I waves. In the patient with cerebral cortex atrophy the output evoked by LM and PA magnetic stimulation is similar. Both techniques evoke a large D wave and no clear I waves but only two very small and delayed peaks (Di Lazzaro et al., 2004b).

Stimulation of the primary motor cortex (M1) by TMS evokes a high frequency discharge in a cluster of cortical pyramidal cells, both in animals and humans. For example, epidural recordings have revealed that a single M1-TMS pulse evokes a complex pattern of discharge in the cortico-spinal (CS) projections, as compared to the single action potential evoked by stimulation of a peripheral nerve. This evoked response has an oscillatory nature, being composed of a series of descending waves that are separated from each other by about 1.5 ms (i.e., ~670 Hz; Di Lazzaro et al., 2012). Based on the hypothetical site of TMS activation of the CS system, the components of this CS volley can be subdivided into three components. The first component (D-wave) originates from CS axon stimulation, the second component (I1-wave) from monosynaptic activation of CS cells, and the later components (late I-waves) is believed to originate from the activation of cortical interneurons at greater anatomical or functional distance from the body of the CS cell. Also, D-, I1- and late I-waves are differentially activated depending on TMS intensity and orientation of the induced electric field (Di Lazzaro et al., 2004a). In neurological patients, an abnormality of this high frequency oscillatory activity has been reported in two rare single cases who had an electrode implanted in the high cervical epidural space for pain treatment (one patient had a stroke; Di Lazzaro et al., 2006) and the other a cerebral cortex atrophy due alcohol abuse (Di Lazzaro et al., 2004b; Figure 2).

In routine SEP diagnostics, the latency and amplitude of low frequency components are evaluated in a large variety of neurological disorders to assess the integrity of the somatosensory system. Peripheral nerve stimulation also elicits HFO activity (>400 Hz; SEP-HFOs) in different relay stations all along the somatosensory pathways (Klostermann et al., 1999). The frequency of these SEP-HFOs is not identical along the whole system, but varies according to the region: very high frequency components (>1000 Hz) are generated in subcortical structures, while slower frequencies (about 600 Hz) are recorded within the cerebral cortex (Klostermann et al., 2002; Hanajima et al., 2004; Insola et al., 2004). A great deal of work has focused on cortical components of upper limb SEPs (for a review, see Curio, 2000). These components decrease during sleep (Yamada et al., 1988; Hashimoto et al., 1996; Halboni et al., 2000) and are powerfully enhanced by arousal (Gobbelé et al., 2000; Restuccia et al., 2004), thus suggesting that they can be utilized to evaluate the activity of arousal-related structures (Restuccia et al., 2004). In idiopathic generalized epilepsy patients, SEP-HFOs are abnormally enhanced with respect to those obtained from healthy volunteers (Restuccia et al., 2007). Furthermore, the same authors observed increased SEP-HFOs in seizure-free childhood and juvenile absence epilepsy (CAE–JAE) patients, whereas they were normal in drug-resistant patients and in all patients with juvenile myoclonic epilepsy (JME), which is an idiopathic epilepsy that is usually drug-resistant. These results might reflect the hyperactivity of arousal-related brainstem structures and this hyperactivity may account for the different clinical outcome among IGE sub-syndromes (Restuccia et al., 2007), i.e., the better prognosis of CAE–JAE with respect to JME patients.

### Visual Cortex Stimulation

Among the peripherally evoked oscillations, it is worth to also mention the steady state visually evoked potentials (SSVEPs). These consist of oscillatory brain responses generated by visual stimuli that are presented at a constant flicker rate. Clinical SSVEPs are usually (although not exclusively) recorded by EEG and their frequency equals the stimulus frequency plus its even harmonics. Typically, SSVEPs demonstrate an excellent signal-to-noise ratio and a distinct spectrum with characteristic peaks (Vialatte et al., 2010). Although they are thought to be generated in the occipital cortex, extracortical sources (e.g., subcortical structures) have been reported, in particular for the low-frequency components of SSVEPs. These oscillatory potentials have been successfully used in cognitive neuroscience as a probe for physiological brain processes (such as visual attention—(Morgan et al., 1996); working memory—(Silberstein et al., 1995); body perception—(Giabbiconi et al., 2016)), as well as in clinical neuroscience to study age-related diseases. In AD, the study of medium and high frequency components of SSVEPs allowed to demonstrate an alteration in the magnocellular pathway of the visual system (Jacob et al., 2002). In PD, which is often associated with visual abnormalities due to retinal dopaminergic deficiency (Stanzione et al., 1992), these evoked oscillations have proven useful for clinical assessment and monitoring of dopaminergic treatments (Tagliati et al., 1996). In epilepsy diagnostics, arguably the most famous application of SSVEPs, intermittent photic stimulation is commonly used for photic driving to study photosensitive epilepsy since it can induce epileptiform waves in the EEG (Fisher et al., 2005).

### TMS-EEG

TMS-EEG allows the co-registration of EEG during TMS, thus providing the opportunity to noninvasively and directly probe the brain’s cortical excitability and time-resolved connectivity, as a function of instantaneous state (Ilmoniemi and Kicić, 2010; Ferreri and Rossini, 2013). Furthermore, analysis of the spectral components of the TMS-evoked EEG responses has revealed that TMS consistently evokes dominant alpha-band oscillations in the occipital cortex (Herring et al., 2015), beta-band oscillations in the parietal cortex, and beta/gamma-band oscillations in the frontal cortex (Rosanova et al., 2009). This has been interpreted to suggest that each cortical area tends to preserve its own natural frequency, even when indirectly engaged by TMS through network effects or when stimulated at different intensities (Rosanova et al., 2009). Moreover, through this methodology, new information has emerged on how ongoing oscillatory activity maps to behavioral output measures. In the healthy motor system, for instance, TMS-EEG has revealed that MEP amplitude (an index of corticospinal excitability) is inversely correlated with spontaneous fluctuations of rolandic alpha power (Zarkowski et al., 2006; Sauseng et al., 2009) and positively correlated with ipsilateral prefrontal beta activity as well as with bilateral centro-parietal-occipital delta activity (Mäki and Ilmoniemi, 2010; Ferreri et al., 2014). Interestingly, in older adults this pattern is only partially preserved, possibly reflecting a compensatory phenomenon to physiological aging (Ferreri et al., 2017). In PD patients who underwent unilateral thalamotomy (ventrolateral nucleus), a preliminary TMS-EEG study demonstrated higher TMS-induced beta amplitudes in the intact hemisphere, relative to stimulation of the AH. This may confirm a significant contribution of the motor thalamus in the facilitation of cortically generated oscillation through cortico-subcortico-cortical feedback loops. Moreover, these patients showed abnormal TMS-induced beta oscillation in the intact hemisphere when compared to young healthy controls (Werf et al., 2006), supporting that TMS-EEG can be a useful technique to non-invasively monitor pathological oscillatory activity in patients.

**Figure 3:**
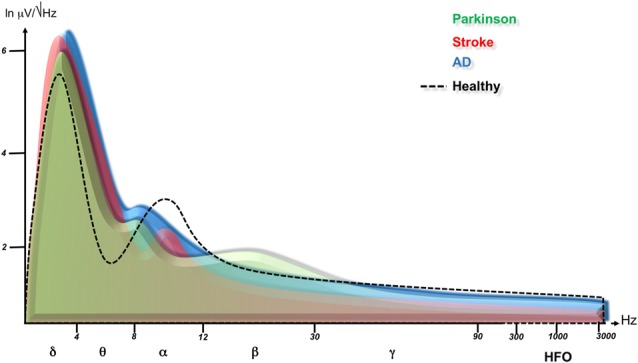
Cortical oscillation changes in neurological disorders. This figure is a summary of published data and shows power spectra after Fast Fourier Transformation are superimposed for healthy subjects (dashed line), Parkinson disease (green, Jenkinson and Brown, 2011; Melgari et al., 2014), stroke (affected hemisphere (AH), red, Tecchio et al., 2005; Graziadio et al., 2011; Assenza et al., 2013b) and Alzheimer disease (blue, Babiloni et al., 2013a,b). Note that neurological disorders cause an increase of slow rhythms, possibly as an attempt to overcome the dysfunction through cortical network synchronization. Degenerative diseases downshift the alpha frequency peak corresponding to a worsening of cognitive performance. Parkinson disease shows a specific beta rhythm increase consistent with an anti-kinetic condition, while the stroke hemisphere produces a reduction of rapid oscillations (alpha, beta, gamma) paralleling with motor performance. Please see the text of the manuscript for more references.

In summary, several neurological conditions of elderly are known to be associated with abnormal oscillatory profiles in specific frequency bands, depending on the disorder (Figure 3). Many of these oscillations normalize with treatment, and/or are predictive of disease progression or recovery. Importantly also, some of these oscillations could be modulated with a functional neurosurgery approach. In the following, we ask the question to what extent existing NIBS protocols may be tailored to interact with these pathological oscillations and possibly also with associated dysfunctions.

## NIBS Modulation of Oscillatory Activities

### Brief Introduction of Main NIBS Techniques

TMS is a non-invasive and painless method to stimulate excitable neuronal tissue with an electric current induced by an external time-varying magnetic field (Rossini et al., 1994). Its key feature is the unique ability to probe changes in excitability, connectivity, as well as global plasticity of intracortical circuits in specific brain areas (particularly motor and visual cortex) or even to induce plasticity by means of non-physiologically induced neuronal activity using repetitive TMS (rTMS) paradigms (Di Lazzaro and Ziemann, 2013). The latter, rTMS protocols were introduced in 1989 using consecutive TMS pulses with a progressively shorter interpulse interval (as short as 10 ms, for a review, see Fitzgerald et al., 2006; Di Lazzaro et al., 2011). For rTMS, a specific set of stimulators is needed to overcome the recharging time and maintain constant TMS output. rTMS has a modulatory effect on cortical excitability which outlasts the stimulation period and can be used in a variety of ways both in motor and non-motor brain regions and with local and nonlocal effects on brain activity. The after-effects of rTMS might relate to activity-dependent changes in the effectiveness of synaptic connections, reflecting plasticity mechanisms of the brain (Di Lazzaro et al., 2011). The majority of research on the effects of rTMS on cortical activity has focused on M1-TMS, as the excitability of this brain region can be easily measured via MEPs amplitude modulation. In terms of the directionality of aftereffects, there is a relative consensus that rTMS frequencies below 1 Hz are mainly inhibitory, while repetition rTMS rates of 5 Hz and beyond are mainly facilitatory at least with regard to the corticospinal motor output. In addition, other patterned protocols such as theta burst stimulation (TBS) or quadripulse stimulation (QPS) have been introduced, which opens new intriguing horizon for TMS (Di Lazzaro et al., 2011). Moreover, in the last two decades, advances in the coupling of TMS with functional imaging techniques has allowed to study the temporo-spatial patterns of local and distal plastic changes in the brain following TMS. This has provided a sensitive means for identifying brain regions where changes in regional neuronal activity correlate with TMS-outcome on motor performance and behavior in both healthy and pathologic conditions. When declined in its specific paradigms—such as rTMS or coupled with different kinds of neuroimaging techniques, TMS has the potential for sophisticated uses to study and/or modify the regional cortical involvement in processes as diverse as motor and cognitive functioning (Rossini et al., 2015; Thut et al., 2017). For example, if the modulation of a target cortex is the objective, TMS can be declined in its repetitive paradigms (see e.g., Di Lazzaro et al., 2011). If the understanding of temporal aspects of neuronal processing or interventions is the main focus of an investigational study, TMS can be coupled with neuroimaging methods with a high temporal resolution such as EEG for guiding TMS or documenting its effects (see e.g., Ferreri and Rossini, 2013). For instance, new EEG/MEG-guided approaches explore the benefits of tuning rTMS to underlying brain oscillations to enhance TMS efficacy, e.g., by triggering to high excitability states or tuning to oscillatory frequencies (Thut et al., 2017).

Along with the TMS methods, transcranial electrical stimulation methods have been developing in the last decades. The first to be introduced was transcranial direct current stimulation (tDCS) about 20 years ago. tDCS requires the application of a weak and constant direct electrical current (1–2 mA) through two or more electrodes placed on the scalp (Priori et al., 1998). The main advantage of tDCS is its ability to achieve relatively long-lasting cortical changes after the stimulation has ended, with the duration of these changes depending on the length of stimulation as well as its intensity. tDCS does not induce activity in resting neuronal networks, but modulates spontaneous neuronal activity (Fritsch et al., 2010). In addition, the amount and direction of its effects critically depend on the previous physiological state of the target neural structures (Antal et al., 2014). Its biological effects include changes in neurotransmitters, effects on glial cells and on microvessels, modulation of inflammatory processes and, most importantly, the subthreshold modulation of neuronal membrane potentials which is capable to vary cortical excitability and activity depending on the direction of current flow through the target neurons (for a review, see Woods et al., 2016). Recently, an additional type of transcranial electrical stimulation method has been introduced, namely transcranial alternating current stimulation (tACS). tACS is able to induce or entrain brain oscillations by causing coherent changes in the firing rate and timing of neuronal populations (Antal and Paulus, 2013). It is capable of modulating perception, cognitive functions, and motor performance (for a review, see Woods et al., 2016). If the stimulation is strong enough, such behavioral effects can be achieved simply through the rhythmic modulation of excitability in the form of alternating “Up” and “Down” states, being this due to alternating relative neural depolarization and hyperpolarization. However, only low-current densities are used in studies in humans and so successful stimulation is often thought to leverage the resonance characteristics of the underlying brain. For this to occur, the stimulation frequency must approximate the natural resonance frequency of local neural circuits, so that spontaneous network oscillations are preferentially entrained (Helfrich et al., 2014b; Guerra et al., 2016). Accordingly, tACS effects tend to be frequency and area selective (Feurra et al., 2011). In the case of sensorimotor cortical areas, convergent evidence suggests a tendency for resonant activity to occur at about 10 Hz and 20 Hz (Hari and Salmelin, 1997). Beta activity, centered on 20 Hz, is focused anterior to the central sulcus and stimulation at or near 20 Hz can thus synchronize the activity of populations of pyramidal neurons so that there is increased corticomuscular coherence at the stimulation frequency (Pogosyan et al., 2009). At present, this represents one of the main field of application of this technique (for a review, see Woods et al., 2016).

### Modulation of Brain Oscillations by NIBS in Healthy Subjects

NIBS techniques reach both cortical and subcortical structures (e.g., through anatomical connections; Ruff et al., 2009) and consequently modulate neural oscillations in both the directly stimulated cortex and the associated network areas/structures (Lang et al., 2005; Fregni and Pascual-Leone, 2007; Zaghi et al., 2010; Fox et al., 2014). Therefore, therapeutic application of NIBS could conceptually rely on the interference with subcortical oscillators through cortical targets (Fregni and Pascual-Leone, 2007). However, NIBS effects on brain oscillations seem to be topographical selective. Indeed, the different frequencies at which transient oscillations can be triggered by single pulses of TMS are a function of cortical site (Rosanova et al., 2009), as is the frequency that can be manipulated by rTMS (Thut et al., 2011; Bortoletto et al., 2015; Romei et al., 2016). This suggests frequency specificity of particular functional networks (in line with some of the studies reviewed above). Similar considerations apply to rhythmic stimulation with tACS (see below). The numerous rTMS studies aimed at establishing modulatory effects on different resting EEG rhythms however obtained inhomogeneous results so far, possibly related to the heterogeneity of the stimulation protocols and the investigated cognitive or behavioral tasks (Okamura et al., 2001; Strens et al., 2002; Klimesch et al., 2003; Schutter et al., 2003; Griškova et al., 2007; Brignani et al., 2008; Fuggetta et al., 2008; Grossheinrich et al., 2009; Thut et al., 2011; Noh et al., 2012; Vernet et al., 2013). The most reliable findings are in favor of changes in delta band activity evoked by stimulating frontal areas, which are the main source of the so-called slow traveling wave of sleep (Ferri et al., 2005; Murphy et al., 2009). Indeed, when a single TMS pulse is applied over these regions, both during sleep (Massimini et al., 2004) and wakefulness (Manganotti et al., 2013), cortical activity in the delta band typically dominates the response. Furthermore, spontaneous delta band activity can be increased by intermittent TBS (iTBS), a rTMS paradigm inducing LTP-like activity (Assenza et al., 2013a; Di Pino et al., 2014) and by frontal 10-Hz rTMS (Griškova et al., 2007) over frontal areas. This suggests that frontal stimulation may trigger and or modify the activation of cortical delta oscillators, perhaps favoring cortical plasticity (Assenza and Di Lazzaro, 2015). However, it is worth noting that none of these attempts leads to a reliable improvement of cognitive performance in healthy individual.

Electric NIBS research has recently developed the use of sine-wave electric current, namely tACS (see above) with the goal of modulating endogenous brain oscillations (Antal and Paulus, 2013). Several intuitive and promising findings have been obtained (reviewed in Herrmann et al., 2013; Frohlich, 2015). However, very little is known about how such stimulation modulates neuronal activity (that in turn guides behavior) in the long run (Veniero et al., 2015). It seems that when tACS is delivered with a stimulation frequency in the alpha band, robustly enhanced spontaneous alpha oscillations are observed after stimulation (aftereffects) in comparison to a sham control (Helfrich et al., 2014b; Vossen et al., 2015). However, the mechanisms of these aftereffect, in particular relative to the correlation between stimulation frequency and endogenous frequency, and whether these effects modulate behavior remain unknown. It is unclear whether the aftereffect on brain oscillations can emerge due to an adjustment of the endogenous frequency to the stimulation frequency (entrainment), or whether it is the endogenous frequency that is modulated in amplitude, although initial results would favor the latter scenario (Vossen et al., 2015). Similarly, convincing results are available for enhancement of gamma oscillations (Helfrich et al., 2014a; Strüber et al., 2014) by gamma-tACS in humans. However, a long-lasting modulatory aftereffect has not yet been achieved in this frequency bands (Nowak et al., 2017). Overall, given the small number of tACS studies, many gaps need to be addressed, including the development of principled, computational models and parameterized experiments to transform speculations about underlying mechanisms into carefully tested hypotheses (Frohlich, 2015).

A final NIBS technique that is promising for modulating EEG activity is stimulation with low-intensity, low-frequency (0–300 Hz) magnetic fields (ELF-MFs). This can induce measurable changes in electrical brain activity and influence neuronal functions such as motor control, sensory perception, and cognitive activities. In healthy humans, Bell et al. (1994) compared the effects of 1.5 and 10 Hz MF, while Cvetkovic and Cosic (2009) analyzed the effects of several ELF-MF frequencies (4, 8.33, 10, 13, 16.6 and 50 Hz) on the power of the corresponding EEG bands. In both cases, spectral analysis demonstrated that specific EEG frequencies can be influenced by stimulation at matching MF frequencies. However, similarly to tACS, the mechanisms of the interaction between ELF-MFs and the brain are unclear (Di Lazzaro et al., 2013).

In the following paragraphs, we critically revise the experiments that have been conducted so far in different patient groups.

### NIBS and Brain Oscillations in Neurological Disorders

#### Parkinson’s Disease and Tremor

The neurophysiological circuit underlying parkinsonian tremor showed to be very stable, in frequency domain, indicating a strong oscillating system (di Biase et al., 2017). However, this circuit can be perturbed by NIBS. rTMS is capable to briefly alleviate PD motor symptoms (Zanjani et al., 2015). One possibility is that rTMS could disrupt the excessive beta activity that is characteristic of cortico-subcortical motor network activity in PD patients, similarly to what is supposed to be the mechanism of action of STN DBS. Accordingly, Doyle Gaynor et al. (2008) analyzed the effects of single pulse TMS on pathological STN oscillations recorded from intracranial electrodes in DBS patients who were on medication. Prior to the stimulation, all but two patients presented excessive 13–35 Hz activity in STN, when the patient were at rest. A delayed, transient beta activity suppression was found after ipsilateral and contralateral M1 and SMA (supplementary motor area) TMS (30–50 single TMS pulses delivered every 5 s). This was observed with sub-threshold as well as supra-threshold TMS, suggesting that changes in oscillatory activity in the STN were centrally driven and not due to peripheral afferent inputs secondary to TMS-evoked muscle responses. No effect on alpha band activity was found, in line with the frequency specificity of the target area/function. Importantly, the authors have found that the duration of the beta activity suppression was of about 400 ms following each single TMS pulse. Future studies should therefore investigate whether this duration can be prolonged when rTMS is used, such as 5 Hz rTMS. Longer-term effects would be required to pave the way for NIBS secondary therapeutic implications.

Krause et al. (2014) used tACS over motor cortex in PD patients to study its effects on brain rhythms and motor performance using MEG and behavioral measures during motor tasks (i.e., static isometric contraction, fast dynamic finger tapping, diadochokinesia). The results revealed that 20 Hz tACS attenuated cortico-muscular coupling in the beta band during static isometric muscle contraction in PD patients. Moreover, the same patterned stimulation reduced amplitude variability during finger tapping. These finding were not present when the PD patients were stimulated with 10 Hz tACS, nor in a control group of healthy subjects. The authors concluded that the clinical improvement was possibly frequency specific because of the pathological motor-cortical synchronization in the beta band (i.e., 13–30 Hz).

Tremor at rest is an invalidating PD sign and typically responds weakly to pharmacological therapies. STN DBS efficacy on PD tremor is well known (Okun, 2012). In a recent tACS study, Brittain et al. (2013) investigated the potential of this technique to reduce tremor in a group of tremor-dominant PD patients by stimulating the motor cortex at tremor frequency. In their experiment, for each patient, the authors identified first the most effective phase lag of motor cortex tACS relative to the peripherally recorded rest tremor, showing that, among patients, the mean phase of peak tremor suppression is −139°. Following this first finding, the patients’ motor cortex was then stimulated by closed-loop phase-locked tACS leading to a 50% tremor intensity reduction, likely through a mechanism of phase cancellation. This supports the notion that central oscillators play a key role in tremor pathophysiology, and represents promising targets for NIBS interventions. The same group investigated also the effectivity of tACS in modulating “physiological tremor” showing that while M1 tACS was able to alleviate postural tremor, it did not affect kinetic tremor in healthy subjects (Mehta et al., 2014).

#### Alzheimer’s Disease

Several groups investigated the effects of NIBS on cognitive deficits, but none of them evaluated the NIBS effects on electrophysiological markers of the disease. In mild to severe AD patients, high-frequency (HF) rTMS (10 Hz or higher) over the prefrontal cortex induces a transient improvement in associative memory, and in naming of actions and objects, possibly due to the compensatory recruitment of contralateral prefrontal and bilateral posterior cortical regions (Cotelli et al., 2006, 2008; Solé-Padullés et al., 2006). In eight AD patients undergoing HF-rTMS (five sessions/week for six consecutive weeks followed by maintenance sessions) applied over Broca and Wernicke areas, right and left DLPFC, or right and left parietal somatosensory association cortex, a significant improvement in the ADAS-cog score was found both after 6 weeks and 4.5 months (Bentwich et al., 2011; Rabey et al., 2013). However, despite these initial promising rTMS results on cognitive AD deficits, and the emerging knowledge regarding the alteration of neuronal oscillatory activity in AD (see “Alpha” Section), no study has yet attempted to examine a possible link of cognitive improvements with restoration of oscillatory activity. A challenging open question is whether NIBS could be rendered more effective by optimizing rTMS parameters in order to restore the affected oscillatory properties at the stimulation site and functionally connected brain networks, which would require simultaneous TMS-EEG/MEG recordings.

#### Stroke

Oscillatory MEPs are a reliable marker of NIBS-induced neurophysiological cortical changes. For instance, iTBS over the damaged cortex in a monohemispheric stroke patient (same patient as reported in “Sensory-Motor Cortex Stimulation” Section) resulted in an increase in corticospinal activity evoked from the damaged cortex, as demonstrated by an amplitude increase of evoked I-waves and by the appearance of a further descending wave (Di Lazzaro et al., 2006). In contrast, the total corticospinal volley evoked from the unaffected motor cortex decreased in this patient by about 40%, demonstrating a decrease in the excitability of the corticospinal output of the hemisphere opposite to iTBS. Other studies have considered changes in spontaneous electrical brain activity that occur after a stroke in relation to influences of rehabilitation and NIBS protocols. A positive correlation has been found between better rehabilitation outcome and greater ERD after different rehabilitation programs (Altenmüller et al., 2009; Fujioka et al., 2012). A MEG study found a correlation between motor recovery and reduction in bilateral post-movement beta-ERS as well as in gamma-ERS of the AH during movement (Wilson et al., 2011). Besides the effects on power, reductions in interhemispheric coherence in the high beta and gamma bands have also been reported after a 12-week rehabilitation training (Pellegrino et al., 2012). It’s worth noting that, so far, only two studies have carried out analysis on the modulation of oscillatory activity by rTMS/tDCS enriched rehabilitation in stroke (Krawinkel et al., 2015). In a single post-stroke aphasic patient, 3 weeks of high frequency rTMS over the AH improved naming and comprehension on a repetition tasks, while increasing functional theta and high beta connectivity between the damaged left inferior frontal gyrus and its controlateral homologous area (Dammekens et al., 2014). Furthermore, anodal tDCS over M1 of the AH, has been shown to enhance alpha-band ERD in chronic stroke patients (Kasashima et al., 2012). A recent meta-analysis suggested a beneficial tDCS effect in stroke rehabilitation (Kang et al., 2016), but the mechanisms behind this effect are not understood. In particular, to the best of our knowledge, a possible link between modulated brain rhythms and clinical improvements has not been explored yet.

#### Epilepsy

The incidence of epilepsy peaks in patients younger than 15 years and in those aged 65 or older (Forsgren et al., 2005). Given this epidemiological data, we included the epilepsy in the present review article, although the neuromodulation experiments reported mostly young adult epileptic patients.

Studies reporting rTMS application in epilepsy are encouraging but need to be interpreted cautiously given their often uncontrolled design (Lefaucheur et al., 2014). In the context of randomized, controlled trials, the antiepileptic effects of active rTMS varied widely from no beneficial effects (Theodore and Fisher, 2007) to significant clinical and electroencephalographic improvement (Fregni et al., 2006; Cantello et al., 2007; Sun et al., 2012). Therefore, availably data still do not provide conclusive evidence in favor or against the efficacy of this emerging treatment modality. Limitation in sample size and lack of a control condition could account for the heterogeneity of published results and for the difficulty of drawing definitive conclusions. Consequently, recommendations for the use of rTMS in epilepsy do not exceed Level C recommendation (“possible efficacy”), at least for focal low frequency rTMS of the epileptic focus.

tDCS has been applied in a few dozens of epileptic patients with the main objective of diminishing seizures and/or electroencephalographic epileptiform activity and evaluating the safety of the procedure (reviewed by San-juan et al., 2015). Fregni et al. (2006) conducted the first exploratory randomized sham controlled study of the effects of tDCS in refractory epileptic patients. Cathodal tDCS over the epileptogenic zone reduced the number of EEG epileptiform discharges and number of seizures immediately after and 15 and 30 days relative to the baseline. In a distinct group of pediatric patients, cathodal tDCS suppressed epileptiform discharges in most of the patients for 48 h, but the effect of a single session on EEG abnormalities was not sustained for 4 weeks. In both these studies, the seizure reduction rate was clinically negligible. Faria et al. (2012) reported that cathodal tDCS produces a reduction in epileptiform activity in pediatric patients with continuous spikes and wave syndrome during slow sleep (CSWS). However, this effect was not observed in an experiment conducted on the same syndrome by Varga et al. (2011). We very recently reported a group of ten adult patients with drug-resistant temporal lobe epilepsy showing a clinically significant amelioration of seizure frequency after ctDCS relative to sham tDCS (Assenza et al., 2017a).

Even if the safety of NIBS has been clearly documented in elderly people (Bikson et al., 2016), the vast majority of the above studies are on young and adult patients. Older patients with epilepsy (>65 years) are very rarely included in the trials protocols of rTMS and tDCS. Moreover, even when they were considered (Rotenberg et al., 2009; Morrell, 2011; Stefan et al., 2012; San-juan et al., 2015), these trials or case reviews did not separately analyze or extrapolate data regarding only elderly patients. Thus, conclusive data about specific or different NIBS effects on young vs. elderly groups of patients is missing, although to the best of our knowledge, results in elderly patients did not differ from those in young adults.

Although there is evidence that tDCS and rTMS reduces epileptiform activity, i.e., impacting on a pathophysiological marker of the disease, current protocols do not evoke suitably strong effects to reduce clinical seizures for application in the clinical context. However, cortical excitability is not only affected by direct stimulation of the cortex, but also by chronic peripheral stimulation of cranial nerves, in particular those with conspicuous and widely diffusing vegetative afferences. Invasive vagal nerve stimulation (iVNS) is an extra-cranial neurostimulation protocol that is FDA-approved as an add-on therapy in patients with drug-resistant partial-onset epilepsy. The stimulator is placed along the cervical branch of the left vagal nerve in the neck. Recently, transcutaneous vagal nerve stimulation (tVNS) has been proposed as a non-invasive alternative to iVNS. tVNS consists of stimulating the auricolar conchae in order to activate the auricular branch of the vagus nerve to then reach the brainstem nuclei, which are responsible for the antiepileptic effect of iVNS. A recent neurophysiology study from our group (Capone et al., 2015) demonstrated that, similarly to what has already been demonstrated for the iVNS (Di Lazzaro et al., 2004c), tVNS produces an increase of intracortical inhibition, namely short-interval-intracortical-inhibition (SICI) as evidenced by the paired-pulse TMS paradigm. Animal studies showed equivalent anti-seizure effects of iVNS and tVNS (He et al., 2013b). A recent human study reported an *in vivo* human evidence that tVNS and iVNS engage the same neural pathways (Assenza et al., 2017b). These results corroborate the hypothesis of a biological similarity between the effects of iVNS and tVNS and warrants further studied aimed at evaluating the clinical efficacy of tVNS in epilepsy. Pilot trials of tVNS in epileptic patients (Stefan et al., 2012; He et al., 2013a) reported an improvement of the number of seizure episodes in about 50% of the patients, but they do not allow conclusive results on efficacy so far because of the small number of recruited patients. Stefan et al. (2012) observed a reduction of seizure frequency and of epileptiform EEG discharges in five of the seven patients who underwent tVNS for 9 months. He et al. (2013a) administered bilateral tVNS for 24 weeks to 15 pediatricepileptic patients, and at the end of the observational period, observed a 50% or more reduction of seizures’ frequency in six patient, with the greatest response registered in the first 2 months. In conclusion, tVNS is a promising technique to replicate iVNS results, but more studies are needed to assess clinical efficacy.

Overall, these studies indicate that NIBS in epilepsy is promising, given the solid evidence base for a potential clinical effectivity, but that it is not yet sufficiently refined to achieve clinically perceptible results. On the other hand, invasive direct cortical stimulation is undoubtedly effective in controlling seizures and epileptiform activity. Indeed, Jacobs et al. (2014) found a clear decrease of HFOs after electrical stimulation (with a diagnostic purpose) during surgical exploration in drug-resistant epileptic patients, and this reduction was not limited to the seizure onset zone. Furthermore, stimulation of the epileptic focus with varying pulse frequencies also led to a significant reduction of seizure activity (Morrell, 2011). This further adds to the arguments that there is a promise also in using transcranial electric or magnetic stimulation techniques to interact with epileptiform activity for clinical purposes. Further studies led by neurologist expert in both epilepsy and NIBS will be necessary to improve tDCS and rTMS efficacy in epilepsy.

### Safety of NIBS Application

To date NIBS techniques are overall considered safe, as they have been used since their introduction without serious collateral effects. However, there are some general and specific points that need to be considered depending on NIBS protocol and the study population (e.g., healthy volunteers or patients). Safety issues on the use of rTMS and tDCS in adults and adolescents are reviewed extensively elsewhere (Rossi et al., 2009; Krishnan et al., 2015; Bikson et al., 2016; Palm et al., 2016) and are summarized below, as a full description of them is beyond the scope of this review article.

A major reason of concern that arises from interfering with brain oscillatory activity and that applies to all NIBS techniques is the possibility of increasing excitability and synchronizing neuronal discharge leading to epileptic activity. Although the risk of seizures is reported to be very low and applies to any protocol increasing cortical excitability (beyond transcranial brain stimulation), it must be taken into consideration especially for protocols delivering high intensity pulses at high frequency capable of inducing neuronal spikes, such as high frequency rTMS (Bae et al., 2007; Pereira et al., 2016). Other NIBS protocol inducing subthreshold depolarization such as tDCS or tACS are less of a concern.

Further frequent side effects common to all NIBS techniques are headache and syncope, usually due to psycho-physical discomfort. High intensity TMS has a risk for impairing hearing that can be mitigated by using earplugs. Heating/ burns must also be considered, especially when TMS is applied over EEG electrodes or with deep brain electrodes, which needs to be mitigated by assessing the compatibility of biomaterials with magnetic and electric fields and reducing the duration of stimulation. tDCS and tACS can provoke skin redness or tingling under the electrodes. Moreover, tACS is known to evoke phosphenes during stimulation that are believed to be of retinal origin (Antal and Paulus, 2013). Finally, contraindications to TMS include cardiac pacemakers or other objects or internal devices that are not compatible with magnetic fields. TMS is also best avoided during pregnancy as insufficient data are available on effects on the fetus. However, a recent review on mothers treated with rTMS for major depression during pregnancy reports no complications to the unborn children (Felipe and Ferrão, 2016).

## Conclusions and Future Directions

Over the last two decades, NIBS techniques have been under intense investigation as to their potential for clinical applications, led by the discovery of the influence of electromagnetic fields on human brain functions and their potential to induce cerebral plasticity. Unfortunately, progress has been limited despite the huge experimental efforts spent to identify NIBS applications that can be used during rehabilitation for additive treatment of motor and linguistic deficits in stroke, Parkinson disease, or for drug-resistant epilepsy, Alzheimer disease and psychiatric disorders. So far, the only FDA-approved clinical use of NIBS is for drug-resistant depression (by rTMS).

Taking into account the numerous clinical studies that have been conducted, the issues of low sample sizes and heterogeneity of protocols might account for the failure of some trials, but cannot explain the overall picture of a lack of proven evidence of NIBS effect in neurological disorders. One clue may reside in the fact that, in most of the clinical trials, the only biological neurophysiological variable targeted by neuromodulation is cortical excitability and its marker, i.e., the amplitude of MEP. This trend is likely explained by cortical excitability being an easy-measurable parameter that is susceptible to modification by NIBS. More recently, the question has been raised whether NIBS, which often uses oscillatory pulses or waveforms, can interact with a specific aspect of human brain activity, namely brain oscillations. Initial results are promising. They show that NIBS in its rhythmic form can entrain EEG activity, and in particular induce specific patterns of oscillatory activity by modulating the power of the rhythms residing in specific areas (e.g., alpha for posterior cortex, beta for motor cortex). Other ideas are based on optimizing the timing of TMS application by aligning it to periods of high excitable states as inferred from EEG measures (e.g., specific EEG phase or power values). Although the induction of a consistent and long-lasting clinical effect, even with multiple stimulating sessions, is far from been proven, the reviewed data illustrates that brain oscillations represent informative and traceable biological markers for pathophysiological conditions in different neurological disorders, and potentially effective targets for NIBS intervention. We find solid evidence for very specific alteration of cortical oscillatory activities in the aforementioned diseases, as well as promise from the few therapeutic trials that started to build on the modulation of these pathological oscillatory activities by NIBS. Research on extrapyramidal movement disorders and epilepsy in particular gathered exemplar evidence on how research on a biological marker of a disease can lead to therapeutic research about the disease itself. In epilepsy, it is foremost the fact that its main EEG biomarker (i.e., epileptiform activity) is also pathognomonic of the disease, which facilitates the design and documentation of interventions in NIBS trials. In addition, even if past clinical results of rTMS and tDCS experiments in epilepsy were overall of variable success, some studies reliably demonstrated the reduction of EEG epileptiform activity. In our opinion, these two factors make epilepsy research one of the most promising fields for working towards clinical NIBS applications using more tailored designs, e.g., considering the patients clinical (type, duration and frequency of seizures) and neurophysiological heterogeneity (position, orientation, frequency and strength of the dipole of the epileptic source). Moreover, in extrapyramidal movement disorders, the modulation of beta activity is a consolidated marker for the success of invasive brain stimulation in PD, and preliminary results of NIBS studies seem promising as to the ability of NIBS to modulate basal ganglia activity.

In conclusion, a large number of research groups are working intensively towards tailoring NIBS to induce long-lasting clinical effect. In parallel, new views on brain oscillations as pathophysiological biomarkers are emerging. These biomarkers vary between disorders, suggesting that pre-set NIBS intervention strategies are unlikely to be the panacea for each patient and disease. We conclude that research should focus on the core of the cerebral disease, i.e., the distortion of cortical activity and its pathophysiological meaning, and tailor NIBS to modulate this activity taking into account the functional neural reserve of the elderly patient and the severity of the disease.

## Author Contributions

GA coordinated the manuscript writing among authors, reassembled the single parts and designed the structure of the review article. FC wrote the part on evoked MEP. LB wrote the part on Parkinson. FF wrote the part of EEG/TMS. GA wrote the part on the Alzheimer’s disease. LF wrote the part on SEP. MM wrote the part of tremor. MP wrote the part on stroke. FR wrote the part on clinical cases. GS wrote the part on dystonia. MT wrote the part on epilepsy. GT and VDL scientifically supervised the work of the group and revised the english language. VDL designed this review article.

## Conflict of Interest Statement

The authors declare that the research was conducted in the absence of any commercial or financial relationships that could be construed as a potential conflict of interest.
