# Corrigendum: Oscillatory Activities in Neurological Disorders of Elderly: Biomarkers to Target for Neuromodulation

**DOI:** 10.3389/fnagi.2017.00252

**Published:** 2017-08-02

**Authors:** Giovanni Assenza, Fioravante Capone, Lazzaro di Biase, Florinda Ferreri, Lucia Florio, Andrea Guerra, Massimo Marano, Matteo Paolucci, Federico Ranieri, Gaetano Salomone, Mario Tombini, Gregor Thut, Vincenzo Di Lazzaro

**Affiliations:** ^1^Clinical Neurology, Campus Bio-medico University of Rome Rome, Italy; ^2^Nuffield Department of Clinical Neurosciences, University of Oxford Oxford, United Kingdom; ^3^Department of Clinical Neurophysiology, Kuopio University Hospital, University of Eastern Finland Kuopio, Finland; ^4^Centre for Cognitive Neuroimaging, Institute of Neuroscience and Psychology, University of Glasgow Glasgow, United Kingdom

**Keywords:** neuromodulation, non-invasive brain stimulation, oscillations, TMS/tDCS, EEG

An author name was incorrectly spelled as Giovanni A. The correct spelling is Giovanni Assenza; the correct surname (family name) is Assenza; the correct name is Giovanni; thus when abbreviated it should be cited as Assenza G.

The authors apologize for this error and state that this does not change the scientific conclusions of the article in any way.

The original article has been updated.

## Conflict of interest statement

The authors declare that the research was conducted in the absence of any commercial or financial relationships that could be construed as a potential conflict of interest.

